# How Do You Know Which Health Care Effectiveness Research You Can Trust? A Guide to Study Design for the Perplexed

**DOI:** 10.5888/pcd12.150187

**Published:** 2015-06-25

**Authors:** Stephen B. Soumerai, Douglas Starr, Sumit R. Majumdar

**Affiliations:** Author Affiliations: Douglas Starr, College of Communication, Science Journalism Program, Boston University, Boston, Massachusetts; Sumit R. Majumdar, Medicine and Dentistry and Pharmacy and Pharmaceutical Sciences, University of Alberta, Edmonton, Alberta. Dr Soumerai is also co-chair of the Evaluative Sciences and Statistics Concentration of Harvard University’s PhD Program in Health Policy.

## MEDSCAPE CME

Medscape, LLC is pleased to provide online continuing medical education (CME) for this journal article, allowing clinicians the opportunity to earn CME credit.

This activity has been planned and implemented in accordance with the Essential Areas and policies of the Accreditation Council for Continuing Medical Education through the joint sponsorship of Medscape, LLC and *Preventing Chronic Disease*. Medscape, LLC is accredited by the ACCME to provide continuing medical education for physicians.

Medscape, LLC designates this Journal-based CME activity for a maximum of 1 ***AMA PRA Category 1 Credit(s)™***. Physicians should claim only the credit commensurate with the extent of their participation in the activity.

All other clinicians completing this activity will be issued a certificate of participation. To participate in this journal CME activity: (1) review the learning objectives and author disclosures; (2) study the education content; (3) take the post-test with a 75% minimum passing score and complete the evaluation at www.medscape.org/journal/pcd; (4) view/print certificate.

**Release date: June 25, 2015; Expiration date: June 25, 2016**

### Learning Objectives

Upon completion of this activity, participants will be able to:

- Define healthy user bias in health care research and means to reduce it
- Assess means to reduce selection bias in health care research
- Assess how to overcome confounding factors by indication in health care research
- Evaluate social desirability bias and history bias in health care research

**EDITORS**

Ellen Taratus, Editor, *Preventing Chronic Disease*. Disclosure: Ellen Taratus has disclosed no relevant financial relationships.

Camille Martin, Editor, *Preventing Chronic Disease*. Disclosure: Camille Martin has disclosed no relevant financial relationships.

Jeanne Madden, PhD, Department of Population Medicine, Harvard Medical School, Boston, Massachusetts. Disclosure: Jeanne Madden has disclosed no relevant financial relationships.

**CME AUTHOR**

Charles P. Vega, MD, Clinical Professor of Family Medicine, University of California, Irvine

Disclosure: Charles P. Vega, MD, has disclosed the following relevant financial relationships: Served as an advisor or consultant for: Lundbeck, Inc; McNeil Pharmaceuticals; Takeda Pharmaceuticals North America, Inc.

**AUTHORS AND CREDENTIALS**

Stephen B. Soumerai, ScD, Professor of Population Medicine, Harvard Medical School and Harvard Pilgrim Health Care Institute; Co-chair, Evaluative Sciences and Statistics Concentration of Harvard University's PhD Program in Health Policy, Harvard University, Boston, Massachusetts; Douglas Starr, MS, Co-director of Science Journalism Program at Boston University, Boston University, Boston, Massachusetts; Sumit Majumdar, MD, MPH, FRCPC, Professor of Medicine, Endowed Chair in Patient Health Management, Faculties of Medicine and Dentistry and Pharmacy and Pharmaceutical Sciences, University of Alberta, Edmonton, Alberta, Canada

Disclosures: Stephen B. Soumerai, Douglas Starr, and Sumit Majumdar have disclosed no relevant financial relationships.

**Editor’s Note:** The purpose of this Editor’s Choice article is translational in nature. It is intended to illustrate some of the most common examples of potential study bias to help policy makers, journalists, trainees, and the public understand the strengths and weaknesses of various types of health care research and the kinds of study designs that are most trustworthy. It is neither a comprehensive guide nor a standard research methods article. The authors intend to add to these examples of bias in research designs in future brief and easy-to-understand articles designed to show both the scientific community and the broader population why caution is needed in understanding and accepting the results of research that may have profound and long-lasting effects on health policy and clinical practice.

Evidence is mounting that publication in a peer-reviewed medical journal does not guarantee a study’s validity (1). Many studies of health care effectiveness do not show the cause-and-effect relationships that they claim. They have faulty research designs. Mistaken conclusions later reported in the news media can lead to wrong-headed policies and confusion among policy makers, scientists, and the public. Unfortunately, little guidance exists to help distinguish good study designs from bad ones, the central goal of this article.

There have been major reversals of study findings in recent years. Consider the risks and benefits of postmenopausal hormone replacement therapy (HRT). In the 1950s, epidemiological studies suggested higher doses of HRT might cause harm, particularly cancer of the uterus (2). In subsequent decades, new studies emphasized the many possible benefits of HRT, particularly its protective effects on heart disease — the leading killer of North American women. The uncritical publicity surrounding these studies was so persuasive that by the 1990s, about half the postmenopausal women in the United States were taking HRT, and physicians were chastised for under-prescribing it. Yet in 2003, the largest randomized controlled trial (RCT) of HRT among postmenopausal women found small increases in breast cancer and increased risks of heart attacks and strokes, largely offsetting any benefits such as fracture reduction (3).

The reason these studies contradicted each other had less to do with the effects of HRT than the difference in study *designs*, particularly whether they included comparable control groups and data on preintervention trends. In the HRT case, health-conscious women who chose to take HRT for health benefits differed from those who did not — for reasons of choice, affordability, or pre-existing good health (4). Thus, although most observational studies showed a “benefit” associated with taking HRT, findings were undermined because the study groups were not comparable. These fundamental nuances were not reported in the news media.

Another pattern in the evolution of science is that early studies of new treatments tend to show the most dramatic, positive health effects, and these effects diminish or disappear as more rigorous and larger studies are conducted (5). As these positive effects decrease, harmful side effects emerge. Yet the exaggerated early studies, which by design tend to inflate benefits and underestimate harms, have the most influence.

Rigorous design is also essential for studying health policies, which essentially are huge real-world experiments (1). Such policies, which may affect tens of millions of people, include insurance plans with very high patient deductible costs or Medicare’s new economic penalties levied against hospitals for “preventable” adverse events (6). We know little about the risks, costs, or benefits of such policies, particularly for the poor and the sick. Indeed, the most credible literature syntheses conducted under the auspices of the international Cochrane Collaboration commonly exclude from evidence 50% to 75% of published studies because they do not meet basic research design standards required to yield trustworthy conclusions (eg, lack of evidence for policies that pay physicians to improve quality of medical care) (7,8).

This article focuses on a fundamental question: which types of health care studies are most trustworthy? That is, which study designs are most immune to the many biases and alternative explanations that may produce unreliable results (9)? The key question is whether the health “effects” of interventions — such as drugs, technologies, or health and safety programs — are different from what would have happened anyway (ie, what happened to a control group). Our analysis is based on more than 75 years of proven research design principles in the social sciences that have been largely ignored in the health sciences (9). These simple principles show what is likely to reduce biases and systematic errors. We will describe weak and strong research designs that attempt to control for these biases. Those examples, illustrated with simple graphics, will emphasize 3 overarching principles:

**1. No study is perfect.** Even the most rigorous research design can be compromised by inaccurate measures and analysis, unrepresentative populations, or even bad luck (“chance”). But we will show that most problems of bias are caused by weak designs yielding exaggerated effects.

**2. “You can’t fix by analysis what you bungled by design” (** **10** **).** Research design is too often neglected, and strenuous statistical machinations are then needed to “adjust for” irreconcilable differences between study and control groups. We will show that such differences are often more responsible for any differences (effects) than is the health service or policy of interest.

**3. Publishing innovative but severely biased studies can do more harm than good.** Sometimes researchers may publish overly definitive conclusions using unreliable study designs, reasoning that it is better to have unreliable data than no data at all and that the natural progression of science will eventually sort things out. We do not agree. We will show how single, flawed studies, combined with widespread news media attention and advocacy by special interests, can lead to ineffective or unsafe policies (1).

The case examples in this article describe how some of the most common biases and study designs affect research on important health policies and interventions, such as comparative effectiveness of various medical treatments, cost-containment policies, and health information technology.

The examples include visual illustrations of common biases that compromise a study’s results, weak and strong design alternatives, and the lasting effects of dramatic but flawed early studies. Generally, systematic literature reviews provide more conservative and trustworthy evidence than any single study, and conclusions of such reviews of the broad evidence will also be used to supplement the results of a strongly designed study. Finally, we illustrate the impacts of the studies on the news media, medicine, and policy.

## Case 1: Healthy User Bias in Designs of Studies of Influenza Vaccination 

This case example describes healthy user bias in studies attempting to compare healthy users of influenza (flu) vaccines with unhealthy nonusers (eg, frail, severely ill) and attributing the differences to the vaccines. Flawed results of poorly designed experiments have dictated national vaccination policies. More rigorous longitudinal studies suggest that national flu vaccine campaigns have not lowered mortality rates in the elderly.

### Background

Selection biases may be the most ubiquitous threat to the trustworthiness of health research. Selection bias occurs when differences between treatment recipients and nonrecipients or control groups (based on such factors as income, race, or health) may be the true cause of an observed health effect rather the treatment or policy itself.

Healthy user bias is a type of selection bias that occurs when investigators fail to account for the fact that individuals who are more health conscious and actively seek treatment are generally destined to be healthier than those who do not. This difference can make it falsely appear that a drug or policy improves health when it is simply the healthy user who deserves the credit (11).

One well-known example is the national campaign in the United States to universally vaccinate all elderly people against the flu. The goal is to reduce the most devastating complications of flu, death and hospitalizations for pneumonia (12). No one disputes the idea that flu vaccines reduce the occurrence and symptoms of flu, but the national campaign was based on the assumption that the vaccines could also reduce the number of pneumonia-related hospital admissions and deaths. This assumption was based on dozens of cohort studies that compared what happened to older patients who chose to get a flu vaccination with what happened to older patients who did not or could not.

**Figure 1:**
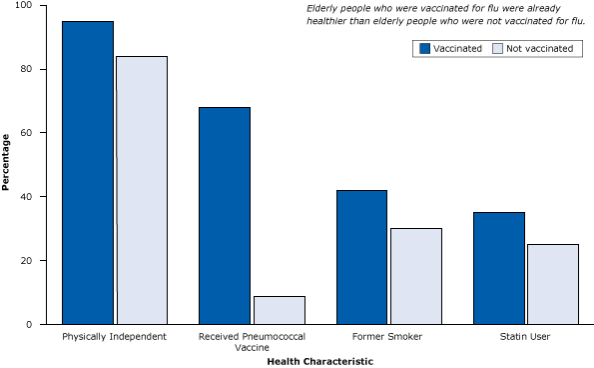
Healthy user bias, a type of selection bias, is demonstrated in a study of 3,415 patients with pneumonia (and at high risk for flu and its complications), where elderly flu vaccine recipients were already healthier than nonrecipients. Figure is based on data extracted from Eurich et al (13). **Table:** 

These cohort studies, however, did not account for healthy user bias. For example, a study of 3,415 people with pneumonia (and at high risk for flu and its complications) illustrated that elderly people who received a flu vaccine were more than 7 times as likely to also receive the pneumococcal vaccine as elderly people who did not receive a flu vaccine (Figure 1). They were also more likely to be physically independent, have quit smoking, and to be taking statins, a medication that improves survival of patients with heart disease, diabetes, and other conditions and prevents heart attacks and strokes among the elderly (13). In short, elderly people who got the flu vaccine already were healthier, more active, and received more treatment than those who did not and so had lower rates of flu-related hospitalization and death during the study period (14).

Characteristic	Vaccinated, %	Not Vaccinated, %
Physically independent	95	84
Received pneumococcal vaccine	68	9
Former smoker	42	30
Statin user	35	25

Healthy user bias is a common threat to research, especially in studies of any intervention where the individual patient can seek out health care and choose to be immunized, screened, or treated (14). This same type of bias is largely responsible for all the many health “benefits” attributed to taking multivitamins, antioxidants such as vitamin C or vitamin E, modest amounts of red wine, vegetarian or low red meat diets, fish oil supplements, chelation therapy, and so on. Most of these interventions, when subjected to randomized trials, show no particular benefits and, sometimes, even harm.

### Weak research designs that do not control for healthy user bias

One of the most common study designs examining the risks and benefits of drugs and other interventions is the epidemiological cohort design, which compares death and disease rates of patients who receive a treatment with the rates of patients who do not. Although seemingly straightforward, this design often fails to account for healthy user bias, especially in studies of health care benefits.

**Figure 2:**
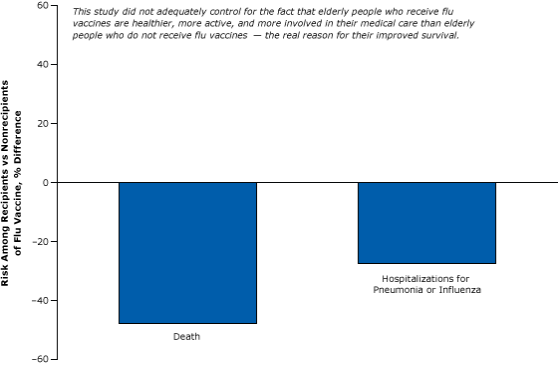
A weak cohort study comparing the risk of death or hospitalization for pneumonia or flu among vaccinated versus unvaccinated elderly: example of failure to control for healthy users. Figure is based on data extracted from Nichol et al (15).

For example, one of many weak cohort studies purported to show that flu vaccines reduce mortality in the elderly (Figure 2). This study, which was widely reported in the news media and influenced policy, found significant differences in the rate of flu-related deaths and hospitalizations among the vaccinated elderly compared with that of their unvaccinated peers (15). Although it controlled for certain easy-to-measure differences between the 2 groups, such as age, sex, and diabetes, it did not account for other more difficult-to-measure “healthy user” factors that affect the well-being of the elderly, such as their socioeconomic status, diet, exercise, and adherence to medical treatments and advice.

The cohort design has long been a staple in studies of treatment outcomes. Because such studies often do not account for people’s pre-existing health practices, they tend to inflate or exaggerate the benefits of treatments (eg, the flu vaccine) while downplaying harms (eg, HRT) (16). In general, we should be skeptical about the benefits of health care interventions (such as the use of drugs or vaccines) reported in cohort studies. On the other hand, the findings of cohort studies related to harms and side effects of medications are often more credible because patients and their physicians do not “choose” to be harmed and tend to avoid known harms. Also, the same healthier people are less likely to have side effects or quit medications. Finally, harms and complications are far rarer than the possible benefits. For instance, whereas the benefits of the flu vaccine can be shown in studies of a few thousand participants, hundreds of thousands of participants might be needed to demonstrate the vaccine’s harms or side effects. For example, Guillain-Barré syndrome occurs in 1 in 500,000 people who receive the flu vaccine.

### Strong research designs that do control for healthy user bias

**Figure 3:**
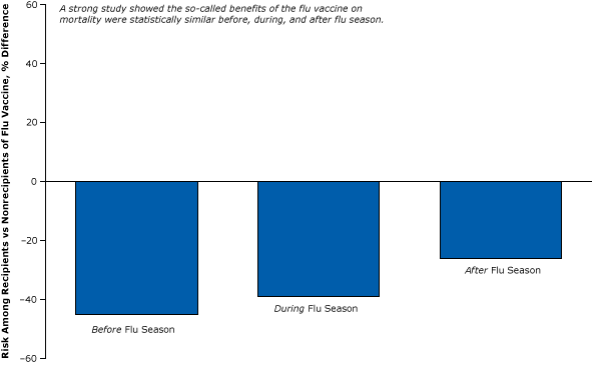
Healthy user bias: a strong controlled study disproving the effects of the flu vaccine on all-cause mortality in the elderly during the flu “off season” (control period). The cohort study compared vaccinated elderly and unvaccinated elderly. Figure is based on data extracted from Campitelli et al (17).

Epidemiological studies that have led to national campaigns have been overturned by subsequent stronger studies. One landmark study (12) found that the fourfold increase in the percentage of elderly people in the United States receiving a flu vaccine during 3 decades (1968–1998) was accompanied not by a decrease, but an increase, in hospitalizations and deaths (Figure 3 in http://archinte.jamanetwork.com/article.aspx?articleid=486407). This does not mean the vaccination is *causing* flu-related deaths or pneumonia. It means the population is getting a bit older and a bit sicker during flu season and the vaccine has little effect among the elderly. This study did not have the healthy user bias found in the previous study because it did not compare health-conscious elderly people who chose to get the flu vaccine with their sicker counterparts who chose not to. Instead, it evaluated whether a marked rise in flu vaccines resulted in fewer deaths over time in the entire population. This study, using a strong design with 30-year trend data, demonstrates the power of pictures — little statistical training is needed to interpret the graph.

A strong, particularly creative study published in 2010 (17) used the same epidemiological design of the weak study illustrated in Figure 2 to show that the so-called benefits of the flu vaccine were statistically equivalent before, during, and after flu season (Figure 3). It is not plausible that the vaccine reduced the flu-related death rate in the spring or summer in the absence of the flu, yet we observe the vaccine “protecting” the elderly all year (17).

The only logical conclusion one can reach from this study is that the benefits during the flu season were simply a result of something other than the effects of flu vaccine — most likely healthy user bias. If fewer vaccinated elders die in the absence of the flu, it is because they are already healthier than unvaccinated elders who may be already too sick to receive a flu vaccination.

Studies with strong research designs that control for selection bias and overturn the exaggerated findings of studies with weak research designs show how weak science in combination with dramatic results can influence the adoption of ineffective health policies. Certainly, greater use of flu vaccine may be reducing the incidence and symptoms of flu. However, the massive national flu vaccination campaign was predicated on reducing the number of flu-related deaths and hospitalizations for pneumonia among the elderly. It could be argued that the funds used for such a campaign could be better spent on developing more effective vaccines or treatments or other methods to reduce the spread of flu.

The news media played a major role in disseminating the misleading results of studies that did not properly take into account the influence of healthy user bias in claims that flu vaccinations could reduce mortality rates and hospitalizations among the elderly. Reuters, for example (Box 1), was unequivocal in its support of a cause-and-effect relationship based on the 2007 report (15) suggesting that flu shots saved lives among the elderly.

**Box 1. Reuters Health, October 3, 2007**

**Flu jab cuts illness and death in elderly**

In a study of relatively healthy elderly HMO members, getting a flu shot significantly reduced the odds of being hospitalized with an influenza-related ailment and of dying. . . . “Our study confirms that influenza vaccination is beneficial for reducing hospitalization and death among community-dwelling HMO elderly over a 10-year period,” said the lead author. . . . Flu vaccination reduced the risk of hospitalization for pneumonia or influenza by 27 percent and reduced the risk of death by 48 percent, the report indicates.

(Excerpted from http://in.reuters.com/article/2007/10/03/us-flu-elderly-idINKUA37737120071003.)

## Case 2: Volunteer Selection Bias in Studies of Health Information Technology

This case example describes volunteer selection biases created by studies that use “volunteer” hospital adopters of health information technology (IT) and noncomparable “laggard” controls (the common design in the field). Volunteer hospitals already tend to have more experienced physicians and healthier patients, which may influence health outcomes more than the intervention does.

The flawed results of these sorts of experiments led to federal health IT initiatives, resulting in trillions of dollars spent on unproven and premature adoption of the technologies and few demonstrated health benefits. RCTs failed to replicate the findings on cost savings and lives saved suggested in the poorly designed studies.

### Background

Researchers often attempt to evaluate the effects of a health technology by comparing the health of patients whose physicians use the technology with the health of patients whose physicians do not. But if the 2 groups of physicians (or hospitals) are different (eg, older vs younger, high volume vs low volume of services), those differences might account for the difference in patient health, not the technology being studied.

Our national investment in health IT is a case in point. Based in part on an influential report from the RAND think tank (18), the 2009 federal stimulus law included a requirement that by 2014 physicians should adopt electronic health records (EHRs) with “decision support” (eg, alerts to reduce the number of duplicate or high-dose drugs). If physicians do not achieve this goal, they will be penalized in the form of reduced Medicare reimbursements. The program is a part of national health care reform and costs trillions of dollars in public and private funds (19). But there is debate about whether health IT can achieve the program’s goals of better health and lower costs. In fact, the RAND think tank has recanted its earlier projections as being overly optimistic and based on less than adequate evidence (20). Furthermore, recent studies (and even the US Food and Drug Administration) are documenting that health IT can lead to the very medical errors and injuries that it was designed to prevent (21,22).

**Figure 4:**
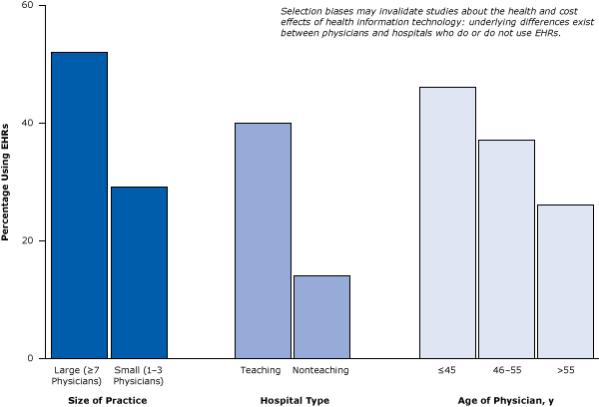
Example of selection bias: underlying differences between groups of medical providers show how they are not comparable in studies designed to compare providers using EHRs with providers not using EHRs. Figure is based on data extracted from Simon et al (23) and Decker et al (24). Abbreviation: EHR, electronic health record. **Table:** 

Let’s examine some studies that illustrate how provider selection biases may invalidate studies about the health and cost effects of health IT. Figure 4 illustrates that underlying differences exist between physicians and hospitals who do or do not use EHRs (23,24). Large physician practices and teaching hospitals are much more likely to use EHRs than are small or solo practices or nonteaching hospitals. Because hospital size and teaching status are predictors of quality of care (with larger hospitals and teaching hospitals predicting higher quality), the 2 factors can create powerful biases that can lead to untrustworthy conclusions. Thus, although studies may associate health IT with better patient health, what they are really pointing out are the differences between older physicians and younger physicians or differences between large physician practices and small physician practices. Such large differences between EHR adopters and nonadopters make it almost impossible to determine the effects of EHRs on health in simple comparative studies. Perhaps as more hospitals adopt EHRs or risk penalties, this type of selection bias may decrease, but that is in itself a testable hypothesis.

Characteristic	Percentage Using Electronic Health Records
**Size of practice**	
Large (≥7 physicians)	52
Small (1–3 physicians)	29
**Type of hospital**	
Teaching hospital	40
Nonteaching hospital	14
**Age of physician, y**	
≤45	46
46–55	37
>55	26

### Weak cross-sectional research designs that do not control for differences in providers

**Figure 5:**
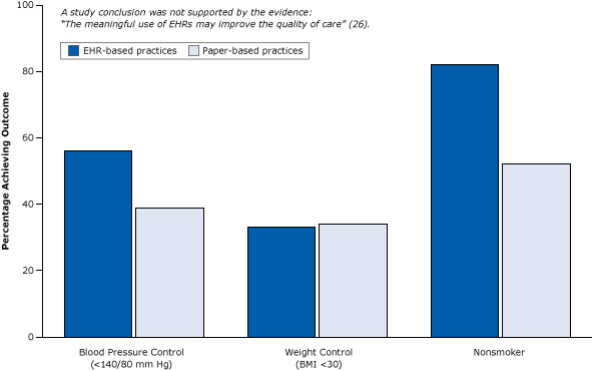
Example of weak post-only cross-sectional study that did not control for selection bias: the study observed differences between practices with EHRs and practices with paper records after the introduction of EHRs but did not control for types of providers adopting EHRs. Note the unlikely outcome for nonsmoker. Figure is based on data extracted from Cebul et al (26). Abbreviations: BMI, body mass index; EHR, electronic health record. **Table:** 

The following example illustrates how a weak cross-sectional study (a simple correlation between a health IT program and supposed health effects at one point in time) did not account for selection biases and led to exaggerated conclusions about the benefits of health IT (25,26). The researchers set out to compare health care sites using EHRs with health care sites using paper records to determine whether patients with diabetes in health care settings with health IT had better health outcomes than patients with diabetes in settings with only paper records (Figure 5). 

Health Outcome	Percentage of Patients Achieving Outcome	
	Electronic Health Record–Based Practice	Paper-Based Practice
Blood pressure control (<140/80 mm Hg)	56	39
Weight control (body mass index <30)	33	34
Nonsmoker	82	52

This weak cross-sectional design would be excluded because of inadequate evidence of the effects of medical services and policies by systematic reviewers adhering to the standards of the international Cochrane Collaboration (27). The study compared outcomes (eg, blood pressure control) of sites with EHRs and sites without EHRs at one point in time *after* the introduction of EHRs but did not provide data on such outcomes *before* the introduction of EHRs; no measure of change was provided. It is virtually impossible to statistically equalize the groups on the hundreds of differences (selection biases) that might have caused differences in blood pressure outcomes; thus, such designs are among the weakest study designs in research attempting to establish cause and effect (9).

The questionable findings of this study suggested that EHRs might not only improve blood pressure control but also reduce smoking by 30 percentage points (Figure 5). (Strong smoking-cessation programs, such as physician counseling programs, studied in rigorous randomized trials have resulted in a 1% to 2% reduction in smoking [28].)

**Figure 6:**
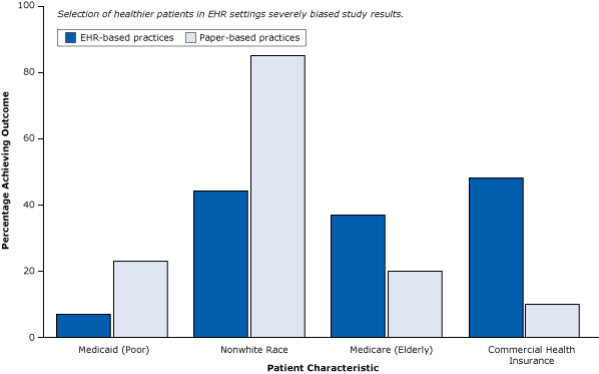
Differences in patient characteristics between EHR-based practices and paper-based practices in a weak post-only cross-sectional study that did not control for selection bias. Abbreviation: EHR, electronic health record. Figure is based on data extracted from Cebul et al (26). **Table:** 

The conclusion of the report — that “the meaningful use of EHRs may improve the quality of care” — is not warranted. Large practices, teaching hospitals, and younger physicians (Figure 4) already deliver better care whether or not they use EHRs. Similarly, even in their own study, the authors found that patients in practices with EHRs had better health care to begin with (Figure 6). They tended to be white, less likely to be poor and rely on Medicaid, and more likely to have commercial health insurance — all indicators of a higher socioeconomic status associated with better care that have nothing to do with EHRs.

Patient Characteristic	Percentage of Patients Achieving Outcome	
	Electronic Health Record–Based Practice	Paper-Based Practice
Medicaid (poor)	7	23
Nonwhite	44	85
Medicare (elderly)	37	20
Commercial health insurance	48	10

Many other kinds of study design (9) can provide better evidence of cause and effect than a post-only cross-sectional design can. Nevertheless, the organization that funded the study, the Robert Wood Johnson Foundation, hailed the results nationally (29), and the news media were exuberant with praise (Box 2).

**Box 2. Science Daily, August 31, 2011**

**Federal Investment in Electronic Health Records Likely to Reap Returns in Quality of Care, Study Finds**

A study . . . involving more than 27,000 adults with diabetes found that those in physician practices using EHRs were significantly more likely to have health care and outcomes that align with accepted standards than those where physicians rely on patient records.

(Excerpted from http://www.sciencedaily.com/releases/2011/08/110831115930.htm.)

### Strong research designs that do control for differences in providers

**Figure 7:**
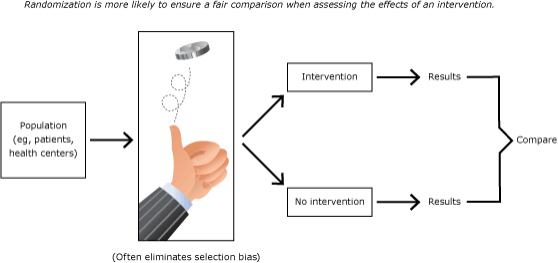
Randomized controlled trial: the “gold standard” of research design.

Given the volunteer selection biases in comparing unlike providers with EHRs and providers without EHRs, what designs can level the playing field and yield more trustworthy results? The “gold standard” of research designs (Figure 7) is the RCT.

This simple design starts with a population (eg, patients, health centers) and uses chance to randomly allocate some centers to the intervention (eg, health IT or not [control]). The researchers then test whether health in the intervention improved more than health in the control. The randomization generally eliminates selection biases, such as facility size or patient age or income. Such designs can reduce bias if they adhere to methodological safeguards, such as blinding patients to their treatment status and randomizing enough patients or centers.

**Figure 8:**
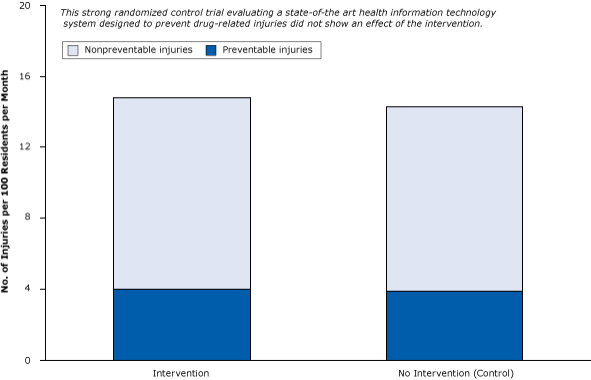
A strong randomized controlled trial of the effect of health information technology on the prevention of drug-related injuries among nursing home residents. Intervention participants received computerized warnings about unsafe combinations of drugs. Figure is based on data extracted from Gurwitz et al (30). **Table:** 

Consider the following randomized control trial involving a state-of-the-art health IT system with decision support in nursing homes (30). By randomizing 29 nursing homes (and 1,118 patients), the researchers controlled for selection biases. The objective of the trial was to examine the effect of computerized warnings about unsafe combinations of drugs to reduce preventable drug-related injuries. The rigorous appraisal of health IT showed that it was ineffective at reducing injuries. Among study patients receiving the health IT intervention, there were 4.0 preventable drug-related injuries per 100 residents per month; among control patients, there were 3.9 preventable drug-related injuries per 100 residents per month (Figure 8). This failure of the health IT intervention was probably due to physicians ignoring most of the warnings, most of which they felt were not relevant to their patients’ health (31). As it often happens in medical research, this strong “negative” study received less attention from the news media than the much weaker but positive studies proclaiming large benefits (5).

Type of Injury	No. of Injuries per 100 Residents per Month	
	Intervention	No Intervention (Control)
Nonpreventable	10.8	10.4
Preventable	4.0	3.9

A single study, no matter how rigorous, should never be considered definitive. The best evidence of what works in medical science comes from systematic reviews of the entire body of published research by unbiased evaluators — after eliminating the preponderance of weak studies. Such a review of hundreds of health IT studies cited a lack of rigorous evidence (Box 3):

**Box 3. Black et al, “The Impact of eHealth on the Quality and Safety of Health Care: A Systematic Overview. PLOS Medicine” (7)**

[T]here is a lack of robust research on the risks of implementing these technologies and their cost-effectiveness has yet to be demonstrated, despite being frequently promoted by policymakers and “techno-enthusiasts” as if this was a given.

Advancements in health IT may well achieve the promised cost and quality benefits, but proof of these benefits requires more rigorous appraisal of the technologies than research to date has provided.

## Case 3: Bias Due to Confounding by Indication in Studies of the Effects of Sedative-Hypnotic Medications on Hip Fractures Among the Elderly

This case example describes *confounding by indication* — biases that plague the field of observational comparative effectiveness of health care treatments. They occur because physicians choose to preferentially treat or avoid patients who are sicker, older, or have had an illness longer. In these scenarios, it is the trait (eg, dementia) that causes the adverse event (eg, a hip fracture), not the treatment itself (eg, benzodiazepine sedatives).

Landmark studies that failed to control for this bias nevertheless influenced worldwide drug safety programs for decades, despite better controlled longitudinal time-series studies that debunked the early dramatic findings published in major journals.

### Background

One of the oldest and most accepted “truths” in the history of medication safety research is that benzodiazepines (popular medications such as Valium and Xanax that are prescribed for sleep and anxiety) may cause hip fractures among the elderly. At first glance, this adverse effect seems plausible because the drugs’ sedating effects might cause falls and fractures, especially in the morning after taking a sleep medication (32). Stronger evidence published 2 decades later debunked this idea (33).

RCTs — in which similar patients are randomized to either treatment or no treatment — are generally too small to detect such infrequent but important outcomes as a hip fracture: each year, less than 0.5% to 1% of the elderly population has a hip fracture (34). Unfortunately, this shortcoming promotes the use of weaker observational studies with cross-sectional designs, which compare health outcomes of people who happen to be prescribed one treatment with people who happen to be prescribed another treatment. Researchers then attempt to adjust for other differences between the 2 groups of people that may actually be responsible for the hip fractures. Confounding by indication is an insidious and powerfully misleading bias that is almost impossible to fix in any study. It occurs because physicians choose or avoid certain treatments for patients who are sicker, older, or have had the illness longer — traits that cause the adverse health event (eg, hip fracture), not the treatment itself.

**Figure 9:**
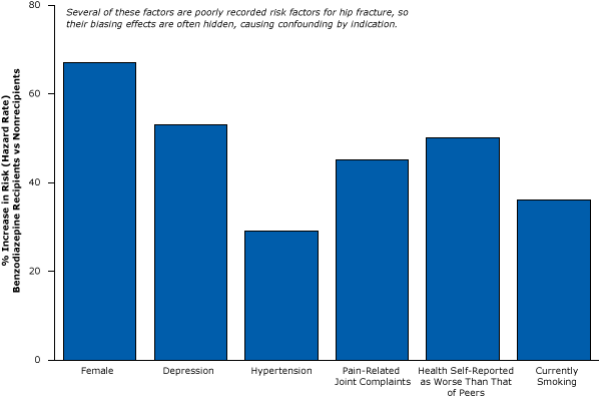
Elderly people who begin benzodiazepine therapy (recipients) are already sicker and more prone to fractures than nonrecipients. Figure is based on data extracted from Luijendijk et al (35). **Table:** 

Confounding by indication may be especially problematic in studies of benzodiazepines because physicians prescribe them to elderly patients who are sick and frail. Because sickness and frailty are often unmeasured, their biasing effects are hidden. Compared with elderly people who do not use benzodiazepines, elderly people who start benzodiazepine therapy have a 29% increased risk for hypertension, a 45% increased risk for pain-related joint complaints (an obvious predictor of hip fractures that is rarely measured in research data), a 50% increased risk for self-reporting health as worse than that of peers, and a 36% increased risk for being a current smoker (Figure 9) (35). Moreover, elderly people prescribed benzodiazepines are more likely to have dementia, a powerful cause of falls and fractures (36). So benzodiazepine users are more likely to fracture their hip even without taking any medication.

Patient Characteristic	Percentage Increase in Risk (Hazard Ratio), Benzodiazepine Recipients vs Nonrecipients
Female	67
Depression	53
Hypertension	29
Pain-related joint complaints	45
Health self-reported as worse than that of peers	50
Current smoker	36

### Weak research designs that do not control for confounding by indication

Almost 30 years ago, a landmark study used Medicaid insurance claims data to show a relationship between benzodiazepine use and hip fractures in the elderly (32). The study has had a worldwide influence on medical practice and helped usher in the modern field of drug safety research. Judging from news media reports and the impact on policy, many people continue to assume that benzodiazepines are a major cause of hip fractures.

**Figure 10:**
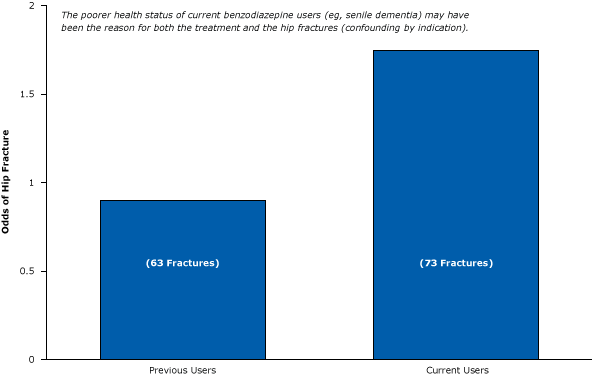
Weak post-only epidemiological study suggesting that current users of benzodiazepines are more likely than previous users to have hip fractures. Figure is based on data extracted from Ray et al (32).

One of several results of this weak post-only epidemiological study showed that current users of benzodiazepines were more likely to fracture their hip than previous users (Figure 10). The authors stated that this comparison permitted them to determine “possible changes in the risk of hip fracture after cessation of drug use.” Unfortunately, they did not actually measure changes in fracture risk after cessation. Instead, they compared people who had already fractured their hip with people who had not (an epidemiological case-control study). They found that hip fractures were more likely to occur among sicker, longer-term recipients of benzodiazepines than among healthier people who took a few pills and stopped. Again, the results seem to have less to do with the drug in question than with the types of people who were prescribed the drug; the poorer health of current users (eg, having senile dementia) may have been the reason for both the treatment and the hip fracture.

The researchers were able to gather little or no data on the sicker, long-term benzodiazepine users from their insurance claims and so could not accurately compare the 2 groups. If they had been able to collect such information, their conclusions may have been different. In short, the researchers could not determine what would have happened if these sicker patients did not receive benzodiazepines.

More than 2 dozen epidemiological studies of hip fractures and benzodiazepines have been published since the original report in 1987 (37). Similar to the flip-flopping results of studies of the risks and benefits of HRT (3), results of these later studies conflicted with each other and with the early reports.

The estimated risks of a fracture shrank over time as investigators did a better job of adjusting for the sicker patients who used benzodiazepines. By the time a more rigorous epidemiological study was conducted that controlled more completely for confounding by indication, the proverbial horse was out of the barn; these investigators demonstrated that the excess risk of benzodiazepines and hip fractures was so small that many considered the risk to be negligible or nonexistent (37).

### Strong research designs that do control for confounding by indication

Case-control studies or “look-back” studies are weak designs for evaluating medical treatments or other interventions because researchers try to draw conclusions when comparing patients whose differences, not treatment, may account for an effect. A stronger research method is the longitudinal natural experiment, in which researchers follow a group over time as their medications or policies that affect them change.

Such natural experiments allow researchers to view multiple points before and after an intervention — to observe a pre-policy trend and a post-policy trend. Rather than comparing different groups of patients at a single point in time, researchers follow patient groups *over* time, to see if a change in medication is accompanied by a change in health. This quasi-experimental research design is called an *interrupted time-series design.* The experiment can be strengthened by following another group of patients who have not experienced the change, a comparison series.

**Figure 11:**
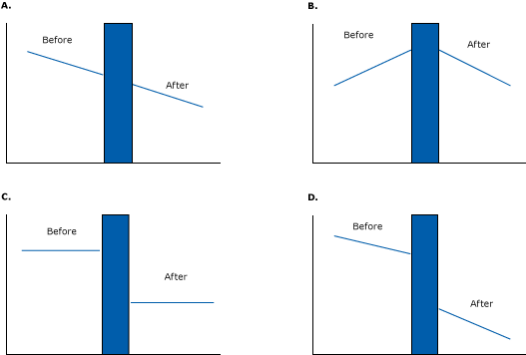
Several examples of effects that can be detected in interrupted time-series studies. The blue bar represents an intervention.

Figure 11 illustrates some of the effects that longitudinal interrupted time-series designs can demonstrate. In Figure 11a, the intervention had no effect on the pre-existing downward trend. If an investigator had simply looked at single data points before and after the intervention (a pre–post design), he or she would mistakenly conclude that the intervention had a large downward effect. But accounting for the baseline trend shows that the intervention probably had no effect.

Figure 11b illustrates a clear downward change from a pre-existing upward trend. A researcher looking at single before-and-after data points would have falsely labeled that intervention a failure (or a success, depending on what was measured) because the downward trend after the program equals the upward trend at baseline. Figure 11c shows a sudden change in level (2 flat lines with a drop caused by an intervention), and Figure 11d shows a pre-intervention downward trend followed by a reduced level and sharper downward trend after the intervention.

These examples illustrate the advantages of graphical data, which can show the true nature of trends. That is not to say that time-series studies never lead to erroneous conclusions. They are just less likely to do so than other designs.

**Figure 12:**
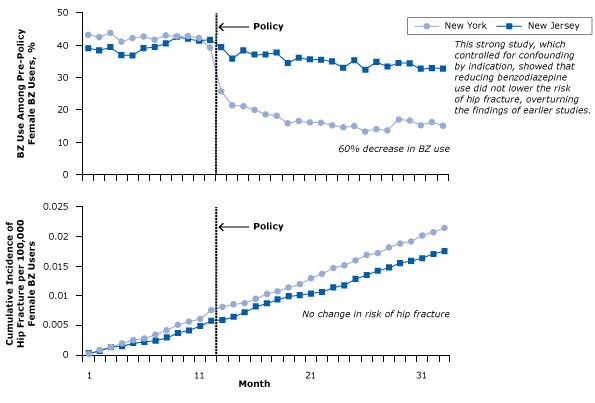
Benzodiazepine (BZ) use and risk of hip fracture among women with Medicaid before and after regulatory surveillance restricting BZ use in New York State. A BZ user was defined as a person who had received at least 1 dispensed BZ in the year before the policy. Figure was adapted from Wagner et al (33). Reprinted with the permission of American College of Physicians, Inc.

In 1989 New York State began to require every prescription of benzodiazepine to be accompanied by a triplicate prescription form, a copy of which went to the New York State Department of Health. State policy makers thought this would limit benzodiazepine use, thereby reducing costs, the prevalence of benzodiazepine abuse, and the risk of hip fracture. (In formulating the policy, policy makers referred to the 1987 landmark study on benzodiazepines and hip fractures [32].) In 2007 researchers examined the effects of the policy with a longitudinal study. The investigators examined health data for tens of thousands of elderly women in New York State, before, during, and after the policy limiting benzodiazepine use had been put into effect. The policy had its intended effect: benzodiazepine use dropped by 60% (Figure 12). The researchers also collected similar data for a control group of elderly women in New Jersey, where no such policy had been put in place, and medication use did not change.

The researchers found that rather than a decrease in the incidence of hip fractures, the incidence continued to rise among New York women throughout the post-policy period; in fact, the incidence was slightly higher in New York than in New Jersey, where benzodiazepine use was constant (Figure 12). Contrary to decades of previous studies, the editors of this study concluded that “[c]ontrolling benzodiazepine prescribing may not reduce hip fractures, possibly because the 2 are not causally related” (33).

Even today, many policies to control benzodiazepine use are based on the early dramatic findings and decades of research that did not control for confounding by indication. Like every other drug or device, benzodiazepines have both benefits and risks, but they probably have no effect on the risk of hip fracture.

The findings of these early and widely cited studies were magnified by the news media, which had a resounding impact on the public, clinicians, and policy makers. Rather than challenging the studies, many reporters simply accepted their conclusions. For example, on the day the 1987 study was published (32), *The New York Times* stated that elderly people who use benzodiazepines were “70% more likely to fall and fracture their hips than those who take no such drugs” and that “thousands of hip fractures could be prevented each year” if use of the long-acting drugs were discontinued. Box 4 shows how *The Washington Post* covered the debunking of the early research, 2 decades later.

**Box 4. The Washington Post, January 15, 2007**

**Study Debunks Sedative’s Link to Hip Fractures in Elderly**

Sedative drugs called benzodiazepines (such as Valium) don’t increase the risk of hip fractures in the elderly, a Harvard Medical School study says. The finding suggests that US federal and state policies that restrict access to these drugs among the elderly need to be re-examined, the study authors added. . . . The policy drastically decreased use of benzodiazepines in New York, and we did not see any decline in hip fracture rates compared to New Jersey.

(Excerpted from www.washingtonpost.com/wp-dyn/content/article/2007/01/15/AR2007011500793.html.)

We have cited several examples of contradictory findings on the association between benzodiazepines and hip fractures among the elderly published several years after misleading observational research was first reported. As it did with the studies on the risks and benefits of HRT, it took many years to debunk the earlier studies that were flawed to begin with and given credence by the news media.

## Case 4: Social Desirability Bias in Studies of Programs to Reduce Childhood Weight

This case example describes bias caused by self-reports of socially desirable behavior (mothers reporting that their children watch less television than they actually watch) that became exaggerated after a controlled trial of a 1-year program to educate mothers to reduce such sedentary activity. Comparing the reports of these mothers with the reports of a control group (not participating in the program) further biased the widely reported findings. The use of unobtrusive computer observations instead of self-reports was a more valid approach.

### Background

There is a widespread bias in health research studies that leads to exaggerated conclusions and could be curtailed through the application of common sense. Researchers often use self-reports of health behaviors by study participants. But if the participants in such a study believe that one outcome is more socially desirable then another (such as avoiding fatty foods or exercising regularly), they will be more likely to state the socially desirable response — basically telling researchers what they want to hear.

**Figure 13:**
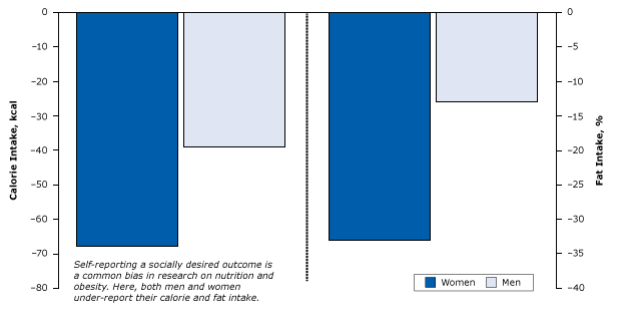
Underreporting of calories and fat consumption due to social desirability among women and men. Figure is based on data extracted from Hebert et al (38). Fat intake was measured as the absolute percentage change for every 1% change in social desirability bias. The zero-line indicates no underreporting. **Table:** 

Some of the more interesting examples of this bias involve studies of obesity and nutrition. A 1995 study showed that both men and women tended to understate their true calorie and fat consumption by as much as 68% in comparison to more objective methods (Figure 13). Women were 2 to 3 times more likely to underreport fat and calorie intake then men (38).

Measure	Underreporting	
	Women	Men
Caloric intake, kcal	−68.0	−38.9
Fat intake, percentage	−33	−13

These women were not lying. They were unconsciously seeing their behavior as conforming to positive societal norms. The principle applies to physicians as well. For example, when asked about their compliance with national quality of care guidelines, physicians overstated how well they did by about 30% in comparison to more objective auditing of their clinical practices. Just like those men and women self-reporting calorie and fat intake, these physicians were not lying or deliberately misleading — they knew what they should be doing and were pretty sure that they were doing it almost all the time (39).

### Weak research designs that do not control for social desirability bias

Even very strong research designs like RCTs can be compromised if the investigators unwittingly tip off the study group to the desired outcomes.

**Figure 14:**
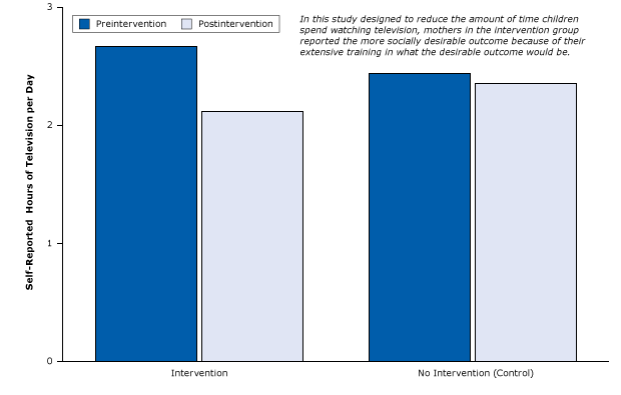
Study that contaminated intervention group by unwittingly tipping parents off to the socially desired outcome: fewer hours of television time per day for children. Figure is based on data extracted from Taveras et al (40). **Table:** 

The following example is one of many published studies that created selection bias due to social desirability. The study was an RCT of a 1-year primary care education program, High Five for Kids, which attempted to motivate mothers to influence their children to watch less television and follow more healthful diets to lose weight (40). After receiving extensive, repetitive training in various ways to reduce television time, mothers in the intervention group were asked to estimate how much less television their children were watching each day. The control group consisted of mothers who did not receive training. Not surprisingly, after the intervention the mothers trained to reduce their children’s television watching reported significantly fewer hours of television watching than mothers in the control group (Figure 14).

Timing	No. of Self-Reported Hours of Television per Day	
	Intervention	No Intervention
Before intervention	2.67	2.44
After intervention	2.13	2.36

Studies with important limitations in design nevertheless can have significant policy implications. On the basis of this study, the High Five for Kids program was declared a success and was a model for an obesity prevention research program in Mexico.

### Strong research designs that do control for social desirability bias

In childhood obesity research, it is difficult to design studies that eliminate social desirability bias. In a comprehensive review of measures of television watching, most studies used self-report (41). But it is possible to use better study designs.

**Figure 15:**
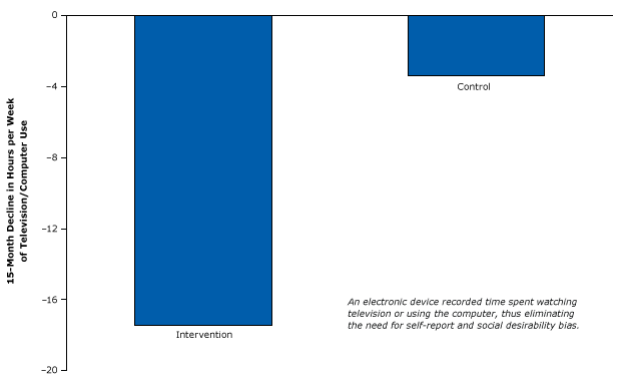
Strong randomized controlled trial design using an electronic device that caused an involuntary reduction in television and computer use. The difference in decline in viewing between the intervention group and control group was significant. Figure is based on data extracted from Epstein et al (42).

In 2008, researchers published a randomized controlled study of an intervention to reduce childhood television and computer use to decrease weight (42). Recognizing biases caused by self-reports and social desirability bias, the investigators installed an electronic device that was used to block about half the household television and computer time of one group of families (the intervention group). The investigators electronically measured the screen time of those families for 15 months and compared it with the screen time of families in a group whose screens were not blocked (control group) during that time. The participants did not know, and were not asked, how much television they were watching, and the researchers did not know which participants belonged to which group. These measures avoided socially desirable self-reporting, making the study results more valid than those in the previous example. The device reduced the amount of time spent watching television and videos by almost 18 hours per week in the intervention group, about 5 times more than the reduction found in the control group (Figure 15). Children in the intervention group also lost more weight than children in the control group.

## Case 5: History Bias in Studies of Hospital Patient Safety Initiatives

This case example describes history bias: uncontrolled pre-existing or co-occurring downward trends in mortality that investigators mistakenly attributed to their national patient safety initiatives. Flawed results from their experiments led to worldwide movements to adopt and entrench ineffective initiatives. In studies of health care and policies, it is essential to graph and display time trends before and *after* the intervention — a fundamental element of time-series studies. Stronger designs using baseline secular trends debunked the early, exaggerated studies, but only after worldwide adoption of the weak initiatives.

### Background

A common threat to the credibility of health research is history bias. History bias can occur when events that take place before or during the intervention may have a greater effect than the intervention itself. An example of this kind of bias took place in a study of an intervention using medical opinion leaders to recommend appropriate drugs to their colleagues for patients with acute myocardial infarction (43).

**Figure 16:**
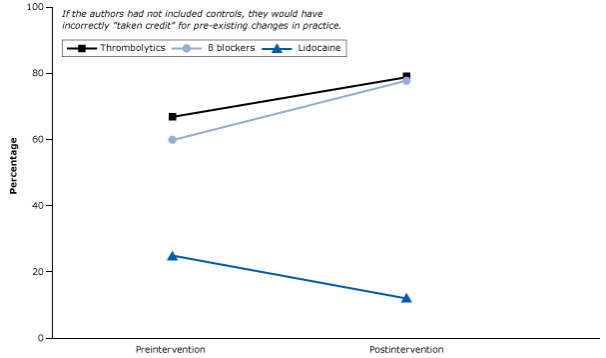
Percentage of acute myocardial infarction patients who received essential life-saving drugs (β blockers and thrombolytics) and a drug linked with increased mortality (lidocaine) in control hospitals before and after an intervention. Figure is based on data extracted from Soumerai et al (43).

Control hospitals (ie, those that did not receive the intervention) still had the desirable changes (Figure 16). These changes were 1) the increased use of 2 medications, β blockers and thrombolytic agents, both of which reduce mortality and 2) a decreased use of lidocaine, the routine use of which is associated with increased mortality (43). The figure illustrates that care improved even without the intervention. In other words, other historical forces were leading to the increased use of effective treatments and the decreased use of harmful drugs.

What could cause such historical biases? This intervention took place during an explosion of research and news media reporting on treatments for acute myocardial infarction that could have influenced the prescribing behavior of physicians. At the same time, the US Department of Health and Human Services launched a national program targeting the drugs in the study, and the American College of Cardiology and the American Hospital Association jointly released new guidelines for the early management of patients with acute myocardial infarction. In the complex environment of health care, policies, and behavior, hundreds of historical events, if not controlled for, could easily account for the “effects” of policies and interventions. Fortunately, the use of a randomized control group in this example accounted for changes that had nothing to do with the study intervention.

### Weak research designs that do not control for history bias

**Figure 17:**
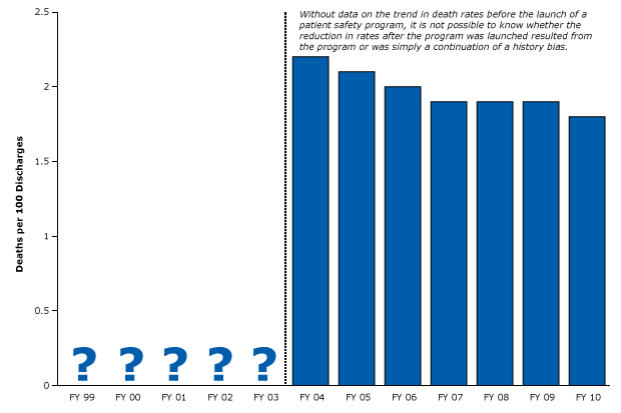
Example of a weak post-only study of a hospital safety program and mortality that did not control for history. Narrow bar shows start of quality of care program. There is no evidence that data are available for the years leading up to the program. The study did not define the intervention period other than to state that planning occurred in 2003. Figure is based on data extracted from Pryor et al (45). Abbreviation: FY, fiscal year. **Table:** 

In 1999, the Institute of Medicine issued a landmark report on how the misuse of technologies and drugs may be causing illnesses and deaths in hospitals throughout the nation (44). Since then, researchers and policy makers have been trying to find ways to improve patient safety. However, the research designed to advance this agenda is often too weak to measure the effects on safety. For example, a recent study was designed to measure the impact of a large patient safety program on death rates in one hospital network (45). The program focused on 6 laudable goals, including reducing the number of adverse drug events, birth traumas, fall injuries, hospital-acquired infections, surgical complications, and pressure ulcers. Unfortunately, the investigators measured mortality rates only after planning and initiating the program (Figure 17), so it is impossible to know whether the reduction in mortality rates resulted from the quality improvement program or from the continuation of pre-existing trends (history bias).

Fiscal Year	Deaths per 100 Discharges
1999	Unknown
2000	Unknown
2001	Unknown
2002	Unknown
2003	Unknown
2004	2.2
2005	2.1
2006	2.0
2007	1.9
2008	1.9
2009	1.9
2010	1.8

No data are available for the years before the hospitals put their program in place. Without that baseline data, such post-only designs cannot provide any realistic assessment of a program’s success (Box 5).

**Box 5. Health Affairs, April 2011**

“The Quality ‘Journey’ At Ascension Health: How We’ve Prevented At Least 1,500 Avoidable Deaths A Year — And Aim To Do Even Better” (45).

Equally common, many pre–post studies have only one measurement before the intervention and one measurement afterward. Such a design is not much different than the weak design of the study illustrated in Figure 17, because we have no idea what would have happened anyway on the basis of the missing pre-existing trend in mortality.

Another example of weak design is a study (46,47) of the Institute for Healthcare Improvement’s (IHI’s) program, the 100,000 Lives Campaign, to prevent hospital deaths in the United States. The campaign consisted of interventions such as deploying rapid response teams to help patients with acute decompensations in the hospital and strategies for preventing life-threatening hospital-acquired infections. As in the study on the patient safety program and hospital death rates (45), the researchers in the study on the IHI campaign measured the trends in death rates only one year before and several years *during* the study period. They created, in essence, a weak pre–post study design with no control group to account for previously occurring changes in deaths that may have had nothing to do with the program (46,47). The IHI issued a press release claiming the program saved 122,300 lives in an 18-month period, which a credulous media repeated (Box 6). But without data on pre-existing trends, IHI’s conclusion that the program saved lives is not tenable.

**Box 6. The Associated Press, June 14, 2006**

**Campaign against hospital mistakes says 122,000 lives saved**

A campaign to reduce lethal errors and unnecessary deaths in U.S. hospitals has saved an estimated 122,300 lives in the last 18 months, the campaign’s leader said Wednesday. . . . “We in health care have never seen or experienced anything like this,” said Dr. Dennis O’Leary, president of the Joint Commission on Accreditation of Healthcare Organizations.

(Excerpted from www.foxnews.com/story/2006/06/14/campaign-against-hospital-mistakes-says-122000-lives-saved-in-18-months/.)

### Strong research designs that do control for history bias

**Figure 18:**
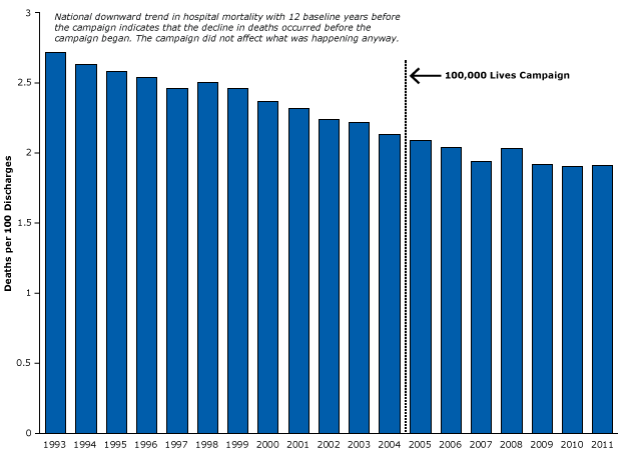
Example of a strong time-series design that controlled for history bias in the Institute for Healthcare Improvement’s 100,000 Lives Campaign. Figure is based on data from the Agency for Healthcare Research and Quality (48). **Table:** 

Does more rigorous evidence support the notion that the 100,000 Lives Campaign actually reduced mortality rates? To investigate that question, we obtained 12 years of national statistics on hospital mortality, longitudinal data from *before* the program went into effect (48). We found that mortality was already declining long before the program began (Figure 18) and that during the program the decline continued at roughly the same rate. These data demonstrate that inpatient mortality in the United States was declining before, during, and after the 100,000 Lives Campaign. The program itself probably had no effect on the trend, yet the widespread policy and media reports led to several European countries adopting this “successful” model of patient safety at considerable costs.

Year	Deaths per 100 Discharges
1993	2.72
1994	2.63
1995	2.58
1996	2.54
1997	2.46
1998	2.50
1999	2.46
2000	2.37
2001	2.32
2002	2.24
2003	2.22
2004	2.13
2005 (Quality of care program began in January 2005)	2.09
2006	2.04
2007	1.94
2008	2.03
2009	1.92
2010	1.90
2011	1.91

Subsequently, several large RCTs demonstrated that many components of the 100,000 Lives Campaign were not particularly effective (49), especially when compared with the benefits reported in the IHI’s press releases.

## Conclusion

Scientists, journalists, policy makers, and members of the public often do not realize the extent to which bias affects the trustworthiness of research. We hope this article helps to elucidate the most common designs that either fall prey to biases or fail to control for their effects. Because much of this evidence is easily displayed and interpreted, we encourage the use of visual data sets in presenting health-related information. To further clarify our message, here (Box 7) is a simple ranking of the ability of most research designs to control for common biases to help readers determine which studies are trustworthy.

**Box 7. Hierarchy of Strong Designs and Weak Designs, Based on Design’s Capacity to Control for Most Biases**

**Table:** 

Hierarchy of Design	
**Strong designs: often trustworthy effects**	
Multiple randomized controlled trials	The “gold standard” of evidence
Randomized controlled trials	A strong design, but sometimes not feasible
Interrupted time series with a control series	Baseline trends often allow visible effects and controls for biases
**Intermediate designs: Sometimes trustworthy effects**	
Single interrupted time series	Controls for trends, but has no comparison group
Before and after with comparison group (single observations, sometimes called “difference in difference” design)	Comparability of baseline trend often unknown
**Weak designs: rarely trustworthy effects (no controls for common biases, excluded from literature syntheses)**	
Uncontrolled before and after (pre–post)	Simple observations before and after, no baseline trends
Cross-sectional designs	Simple correlation, no baseline, no measure of change

Further guidance on research design hierarchy is available (50).

These design principles have implications for the tens of billions of dollars spent on medical research in the United States each year. Systematic reviews of health care intervention studies show that half or more of published studies use weak designs and are untrustworthy. The results of weak study design are flawed science, misconstrued policies, and potentially billions or trillions of wasted dollars.

This article and these case reports barely break the surface of what can go wrong in studies of health care. If we do not learn and apply the basics of research design, scientists will continue to generate flip-flopping studies that emphasize drama over reality, and policy makers, journalists, and the public will continue to be perplexed. Adherence to the principles outlined in this article will help users of research discriminate between biased findings and credible findings of health care studies.

## Figures and Tables

**Table:** 

**1. The activity supported the learning objectives.**				
**Strongly Disagree**				**Strongly Agree**
1	2	3	4	5
**2. The material was organized clearly for learning to occur.**				
**Strongly Disagree**				**Strongly Agree**
1	2	3	4	5
**3. The content learned from this activity will impact my practice.**				
**Strongly Disagree**				**Strongly Agree**
1	2	3	4	5
**4. The activity was presented objectively and free of commercial bias.**				
**Strongly Disagree**				**Strongly Agree**
1	2	3	4	5

## Post-Test Information

To obtain credit, you should first read the journal article. After reading the article, you should be able to answer the following, related, multiple-choice questions. To complete the questions (with a minimum 75% passing score) and earn continuing medical education (CME) credit, please go to http://www.medscape.org/journal/pcd. Credit cannot be obtained for tests completed on paper, although you may use the worksheet below to keep a record of your answers. You must be a registered user on Medscape.org. If you are not registered on Medscape.org, please click on the "Register" link on the right hand side of the website to register. Only one answer is correct for each question. Once you successfully answer all post-test questions you will be able to view and/or print your certificate. For questions regarding the content of this activity, contact the accredited provider, CME@medscape.net. For technical assistance, contact CME@webmd.net. American Medical Association's Physician's Recognition Award (AMA PRA) credits are accepted in the US as evidence of participation in CME activities. For further information on this award, please refer to http://www.ama-assn.org/ama/pub/about-ama/awards/ama-physicians-recognition-award.page. The AMA has determined that physicians not licensed in the US who participate in this CME activity are eligible for ***AMA PRA Category 1 Credits*** ™. Through agreements that the AMA has made with agencies in some countries, AMA PRA credit may be acceptable as evidence of participation in CME activities. If you are not licensed in the US, please complete the questions online, print the AMA PRA CME credit certificate and present it to your national medical association for review.

## Post-Test Questions

### Article Title: How Do You Know Which Health Care Effectiveness Research You Can Trust? A Guide to Study Design for the Perplexed

**CME Questions**

1. Which of the following statements regarding healthy user bias in clinical research is ***most*** accurate?
  - It refers to the fact that healthy people are often excluded from clinical trials
  - It refers to the fact that healthy people actively seek treatment, including that available in clinical trials
  - The best means to reduce healthy user bias is to use an epidemiologic cohort study design
  - It is not possible to mitigate against healthy user bias in cohort studies
2. All of the following factors led to an overestimation of the efficacy of electronic health records (EHRs) in improving patient outcomes ***except:***
  - Selection bias in larger health centers using more EHRs
  - Selection bias in more academic centers using more EHRs
  - Selection bias in younger physicians using more EHRs
  - Testing of EHRs in randomized controlled trials
3. Which of the following methods is the ***best*** means to overcome confounding by indication in cohort studies?
  - An interrupted time-series study design
  - Confirmation of study end points by patients themselves
  - Confirmation of study end points by regional or national databases
  - Elimination of a random half of the study population during study analysis
4. Which of the following statements regarding other types of bias in clinical research is ***most*** accurate?
  - Social desirability bias occurs when researchers alter their outcomes to reflect preferred results
  - Social desirability bias occurs in studies of the general public, but not in research of physicians' behavior
  - History bias refers to trends preceding an intervention that can affect a study outcome more than the intervention itself
  - Evaluating trends in the projected outcome before the intervention does not reduce history bias

**Evaluation**
